# Experimental evidence associates burrowing behavior of *Castalia ambigua* (Bivalvia: Hyriidae) with shell shape and density

**DOI:** 10.1002/ece3.10357

**Published:** 2023-07-25

**Authors:** Gisele do Carmo Reis, Diego Simeone, Colin Robert Beasley

**Affiliations:** ^1^ Laboratório de Conservação da Biodiversidade e das Águas, Instituto de Estudos Costeiros Universidade Federal do Pará Bragança Brazil; ^2^ Laboratório de Bioestatística Instituto Tocantinense Presidente Antônio Carlos Bragança Brazil

**Keywords:** abundance, Amazonia, freshwater bivalve, morphology, movement, sediment

## Abstract

Information on freshwater mussel behavior in the sediment is scarce worldwide, especially in the Amazon. Laboratory experiments were used to measure the responses of the single mussel species *Castalia ambigua* in relation to combinations of two co‐occurring different morphotypes (*Morphotype I* with an elongated shell and *Morphotype II* with a rounded shell), and three different densities (four, eight, and 16 mussels). Horizontal movements (cm) were calculated by summing changes in the position of each specimen and the shell exposure at the sediment–water interface was obtained by measuring (mm) the exposed part of the shell. *Castalia ambigua* presented different patterns of shell exposure and horizontal movements linked with shell shape and density. *Castalia ambigua Morphotype I* remained less exposed with 4 mussels. In contrast, this morphotype was more exposed and tended to aggregate in treatments with 8 and 16 mussels, similar to observations of *Morphotype II* at all densities. *Morphotype II* is mainly found in low hydrodynamic energy habitats, suggesting that patches with high densities may stabilize the substrate around the shells of *Morphotype I*, which is associated with high hydrodynamic energy habitats. We suggest that these patterns may be associated with intrinsic factors of the species, such as reproduction, sexual dimorphism and feeding. Moreover, additional studies using other mussel species belonging to the families Hyriidae and Mycetopodidae are important, since the behavior of these mussels in the sediment may provide useful information on their functional roles in river ecosystems.

## Graphical abstract

Our results suggest a differential response in movement and shell exposure of the freshwater mussel *Castalia ambigua* to density in the two morphotypes: the morphotype associated with high hydrodynamics is more exposed and with higher mussel densities and the morphotype associated with low hydrodynamic energy is more exposed at all densities. We suggest that higher densities may provide stability and allow greater exposure even in high hydrodynamic energy habitats.

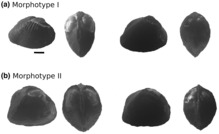

## 1 INTRODUCTION

Most freshwater mussels are endobenthic and use their muscular foot and shell to burrow into the sediment (Allen & Vaughn, 2009; Christian et al., 2020). This behavior may promote substrate destabilization through bioturbation, which modifies the benthic habitat (Vaughn & Hakenkamp, 2001). For example, mussels may mix different sediment layers, altering their physical, chemical and microbial properties (Boeker et al., 2016; Sansom et al., 2022; Simeone et al., 2021a), and thus the abundance of co‐occurring species (Martinski & Woolnough, 2023; Simeone et al., 2021b).

The burrowing behavior of individual mussel species may vary with season and reproductive cycle (Amyot & Downing, 1998), flow regime (Schwalb & Pusch, 2007), substrate composition (Eissenhauer et al., 2023), and mussel density and shell shape (Allen & Vaughn, 2009). Of particular interest for this study, the mussel shell shape is strongly associated with habitat features in temperate rivers (e.g., hydrodynamics and sediment composition; Hornbach et al., 2010; Watters, 1994). These associations are likely linked with horizontal and vertical movements of mussels (Schwalb & Pusch, 2007). Nonetheless, information about these relationships is scarce in the Neotropics. The association between hydrodynamics and shell shape of *Castalia ambigua* Lamarck, 1819 in the eastern Amazon was demonstrated by Simeone et al. (2022) but there is no information on how morphological differences may influence *C. ambigua* behavior in the sediment.

**FIGURE 1:**
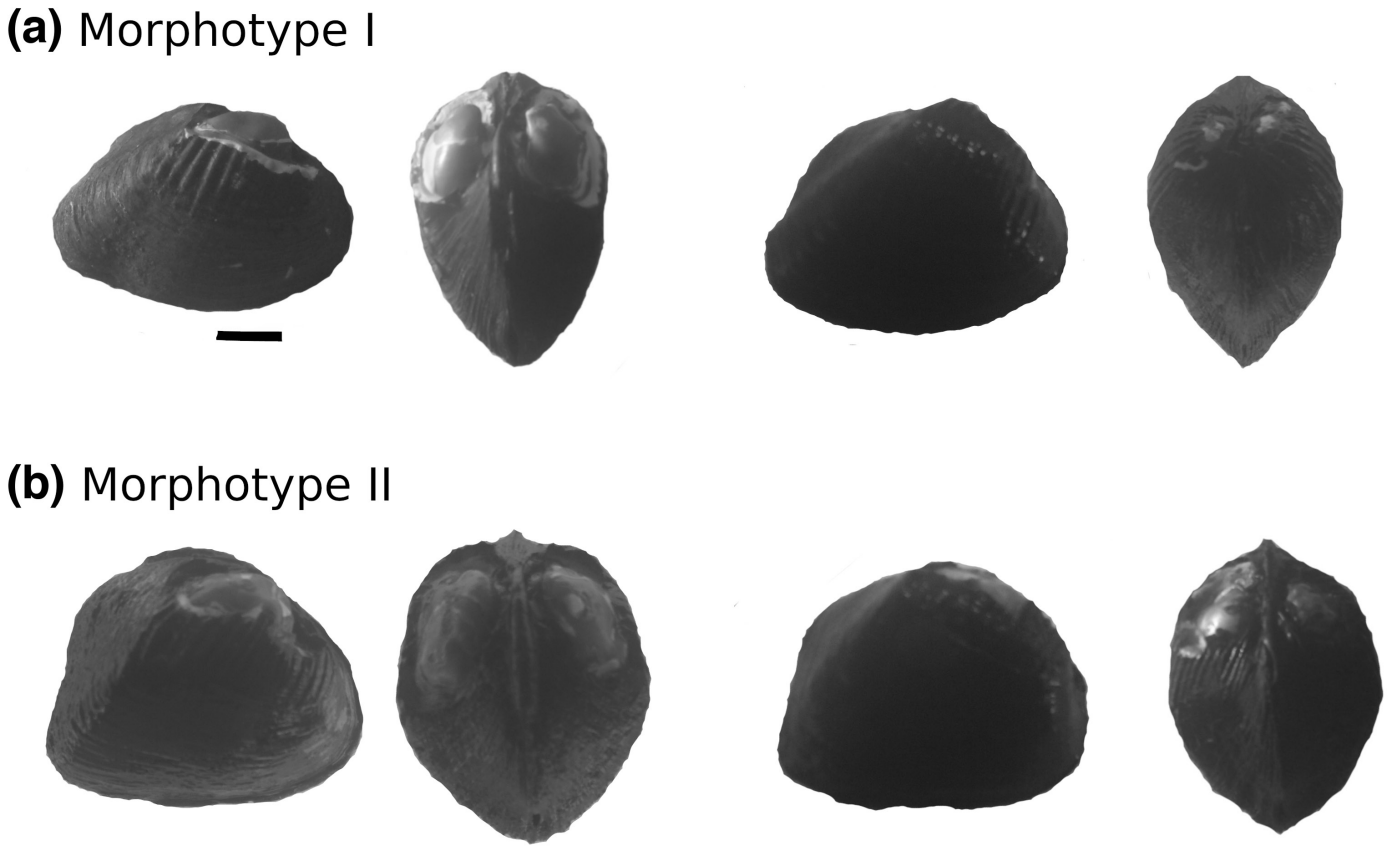
Specimens of *Morphotypes I* and *II* of *C. ambigua* found in the Caeté River used in the burrowing behavior experiments. Scale bar = 1 cm.

South America has approximately 168 native species of freshwater mussels, with species of the genus *Castalia* Lamarck, 1819 (Unionida: Hyriidae) distributed across the main river basins (Olivera‐Hyde et al., 2020). In particular, the species *C. ambigua* is found in the Amazon, Paraguay, and Paraná River basins and has a restricted distribution in the Uruguay River basin (Pereira et al., 2014). In addition, this species is mainly associated with areas of low hydrodynamic energy in river margins with poorly compacted sandy substrates and low silt deposition (Simeone et al., 2021c). Molecular and linear morphometric analyses demonstrated that *C. ambigua* in the upper Paraguay River has phenotypic variation expressed as two morphotypes: one laterally compressed and another inflated (Olivera‐Hyde et al., 2020). In the eastern Amazon, Simeone et al. (2022) using outline morphometric analyses, observed two co‐occurring morphotypes of *C. ambigua* in the Caeté River (Figure 1), that are shaped by sexual dimorphism in habitats with reduced hydraulic energy (Reynolds number of 855 ± 94.6): the first morphotype has an elongate posterior margin and is laterally compressed (male specimens), and the second has rounded anterior–posterior margins and is laterally inflated (female specimens). On the other hand, both morphotypes tended to be similar in areas with high hydrodynamics (Reynolds number of 1604 ± 88.9): elongate posterior margin with the anterior margin laterally inflated. In addition, an intermediate shape was observed in areas with Reynolds number around 1063 ± 68.3 (Simeone et al., 2022). A molecular phylogenetics study has confirmed that only a single species of *C. ambigua* occurs in the Caeté River (Santos‐Neto et al., 2016).

In the Amazon, recent studies have described the effect of *C. ambigua* density (Simeone et al., 2021a), and their filtration and biodeposition activities, on associated macroinvertebrates and water quality (Simeone et al., 2021b). However, the lack of information on horizontal and vertical movements of mussels leads us to ask: How does mussel burrowing behavior vary according to density and shell shape in the Amazon? Thus, the present study, using laboratory experiments, aimed to measure the responses of *C. ambigua* in relation to combinations of two different shell morphotypes and three different densities.

## 2 METHODS

### 2.1 Mussel sampling

**FIGURE 2:**
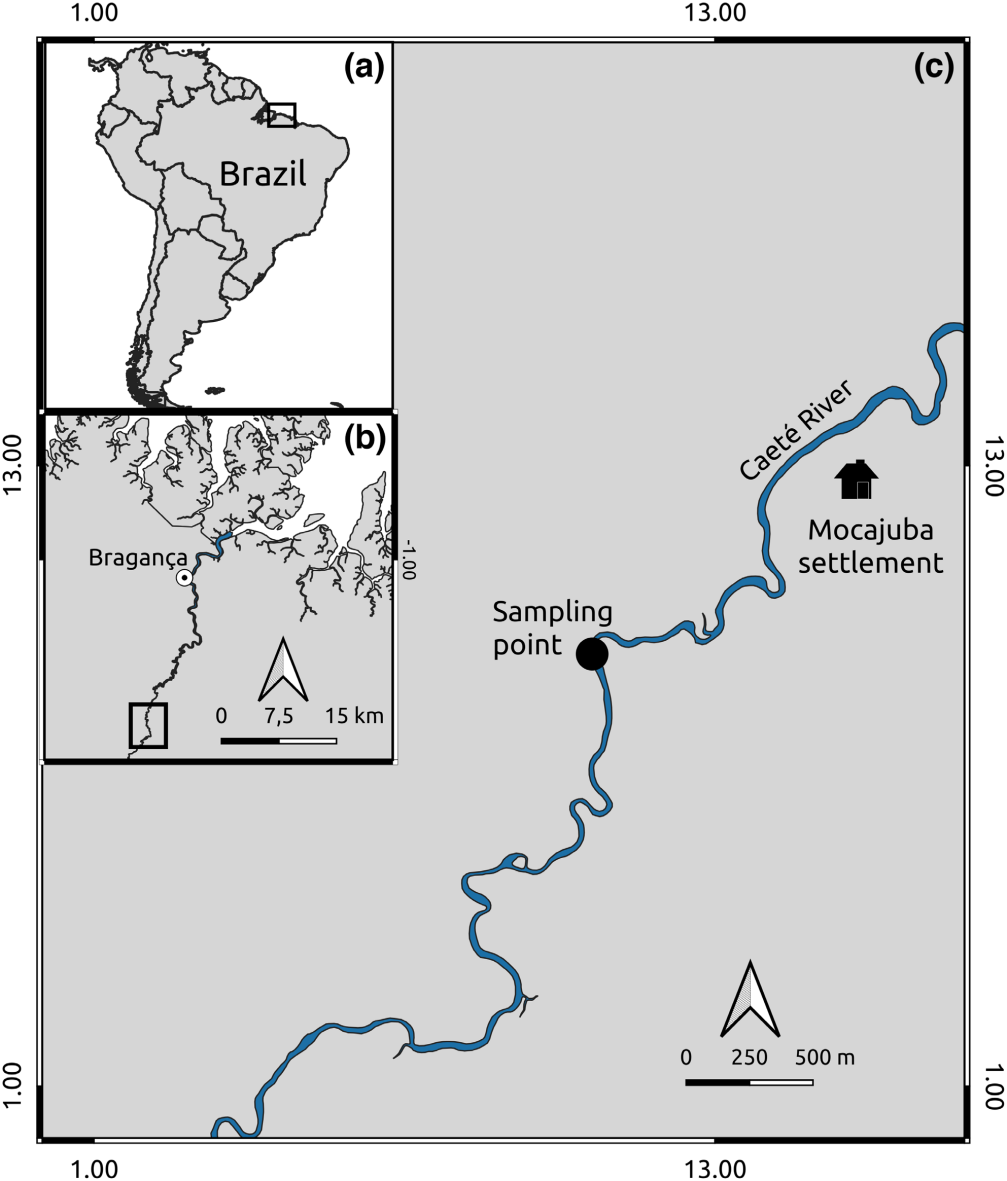
Location of the Caeté River in northern Brazil (a), eastern Amazon (b), approximately 30 km upstream of the city of Bragança, with the single site used for mussel sampling (c).

We collected the specimens of *C. ambigua* used in the laboratory experiment in November 2019 from the Caeté River, a morphologically unaltered alluvial lowland river, approximately 150 km long with a sixth‐order basin, located in northeastern Pará state, in the eastern Brazilian Amazon (Figure 2). Prior to mussel sampling, we collected sediment and water from the Caeté River, which were used to regularly replace the water during the experiments. At a single site (Figure 2c), we collected specimens of *C. ambigua* excavating the sediment by hand to a depth of 15 cm. In this study, we used the two distinct morphotypes described in Simeone et al. (2022), which represent ecophenotypic variation and/or sexual dimorphism (Figure 1): the first with an elongate posterior margin and laterally compressed shells, which we called *Morphotype I*; the second with rounded anterior–posterior margins and laterally inflated shells, which we called *Morphotype II*. Fifty individuals of *C. ambigua* were collected: 26 individuals from *Morphotype I* (mean length of 43.9 ± 2.2 mm) and 24 individuals from *Morphotype II* (mean length of 38.1 ± 1.5 mm). All mussels were placed in a container with a sediment layer and water from their habitat in the Caeté River, transported to the laboratory and maintained in aerated aquarium.

### 2.2 Procedures in the laboratory and experiment

Prior to the experiments, we cultivated microalgae in the laboratory as food for the mussels, using a liquid NPK (nitrogen, phosphorus and potassium) gardening supplement, which resulted in excellent microalgal growth (Simeone et al., 2021b).

For the experiments, we prepared microcosms equipped with aerators that simulated a water current velocity of 0.2 m/s, which is the average velocity found in the habitats of *C. ambigua* in the Caeté River (Simeone et al., 2021c). Each microcosm contained water from the Caeté River and a sediment layer composed of sand and silt, approximating the sediment composition of the Caeté River (Simeone et al., 2021c).

For the experiments, we used three different Morphotype treatments:

- M_I_ – *Morphotype I* of *C. ambigua*
- M_II_ – *Morphotype II* of *C. ambigua*
- M_I+II_ – *Morphotype I* + *Morphotype II in equal proportions*

We combined each morphotype treatment with three different mussel densities (4, 8, and 16 mussels; hereafter D_4,_ D_8_ and D_16_). Three microcosms (replicates) were maintained simultaneously for each combination of morphotype and density: for example, three microcosms for M_I_ with D_4_, and so on. All microcosms were maintained for 6 days, with three observations made on days two, four, and six. A total of 81 observations (9 combinations × 3 replicates × 3 days of observations) were obtained for the experiment, similar to that obtained by Allen and Vaughn (2009). Between each set of combinations, we replaced the water in the microcosms.

We marked each mussel on the central and posterior parts of the shell using shellfish glue‐on tags. Thus, horizontal positions and shell exposure could be recorded for each set of observations. Horizontal movements (cm) were calculated by summing changes in the position of each mussel, using 5 × 5 cm grids that were placed over the microcosms without touching the water. The average values of shell exposure at the sediment–water interface were estimated by measuring (mm) the exposed part of each mussel shell using a caliper (precision of 0.05 mm).

### 2.3 Statistical analysis

All statistical analyzes were carried out in GNU R (R Core Team, 2022). We used a two‐way analysis of variance (ANOVA) with repeated measures to test whether the burrowing behavior of *C. ambigua* varied between the three mussel densities and among the two morphotypes. The treatments Morphotype (M_I_, M_II_ and M_I+II_) and Density (D_4_, D_8_ and D_16_) were used as fixed effects. The random effect was included with the factor Replicate (*n* = 3 replicates) nested in the factor Day (days of observations: two, four and six) as a repeated measure. Mixed‐effects models provide a flexible and powerful tool for modeling the within‐group correlation often present in repeated measures data (Pinheiro & Bates, 2000; Zuur et al., 2009).

## 3 RESULTS

**FIGURE 3:**
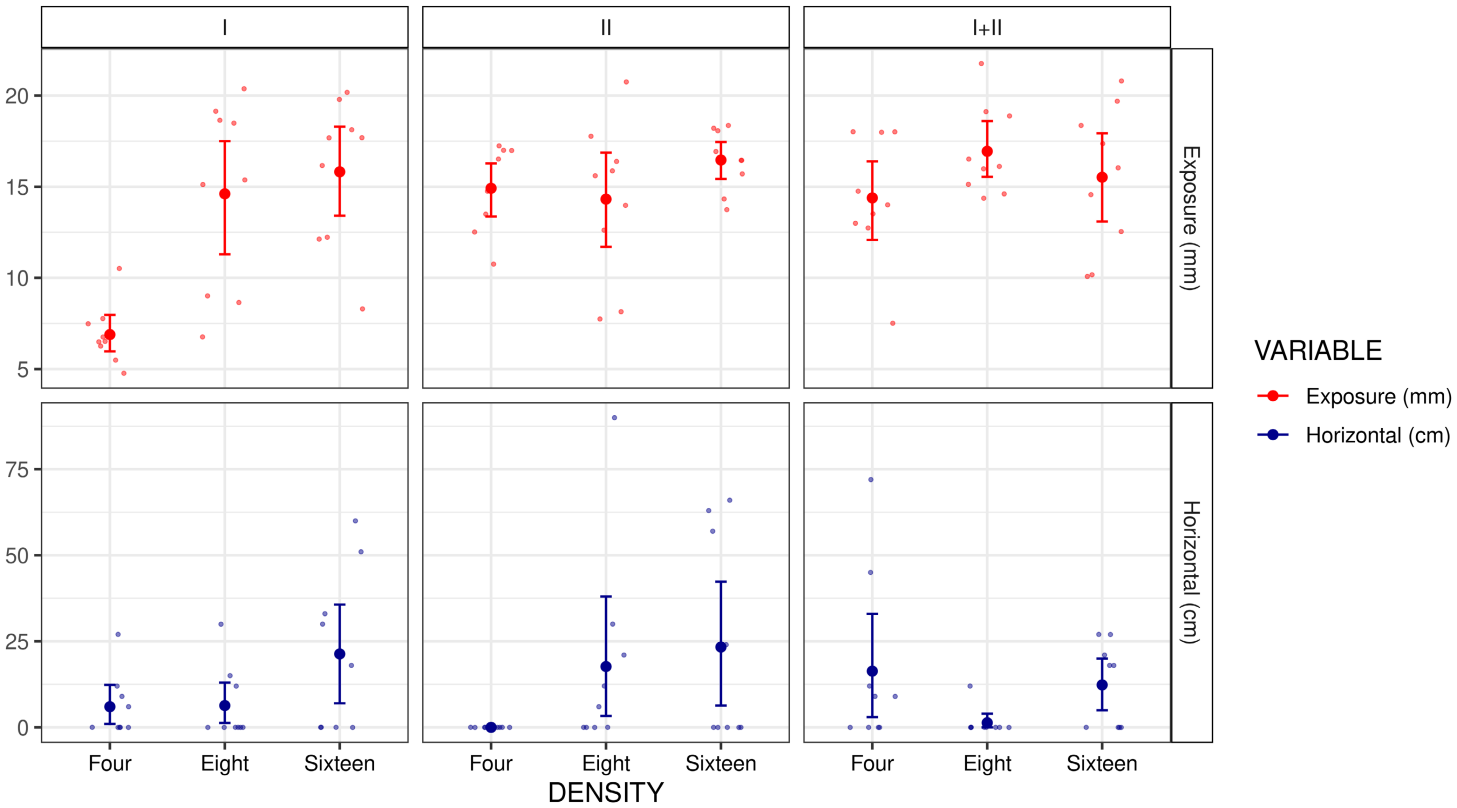
Mean (± robust bootstrapped 95% confidence interval) for shell exposure (mm) and horizontal movement (cm) of mussels among treatment combinations of shell morphotype (M_I_, M_II_, and M_I+II_) and mussel density (four, eight, and sixteen mussels per microcosm). Smaller points represent the raw data.

*Castalia ambigua* presented a significantly high shell exposure for M_I_ in treatments D_8_ and D_16_ but not for D_4_ (Table 1; Figure 3). On the other hand, M_II_ and M_I+II_ presented high shell exposure for all mussel densities (Figure 3). For horizontal movements, the Morphotype effect and the Morphotype by Density interaction were not significant (Table 1; Figure 3). However, both morphotypes showed a marginally significant increase in horizontal movements in the treatment D_16_ for M_I_ and in the treatments D_8_ and D_16_ for M_II_ (Table 1; Figure 3).

**TABLE 1:** ANOVA summaries for shell exposure (mm) and horizontal movement (cm) among treatment combinations of shell morphotype (M_I_, M_II_ and M_I+II_) and mussel density (four, eight, and sixteen mussels per microcosm).

Source of variation	Exposure (mm)		Horizontal (cm)	
	*F*	*p*	*F*	*p*
Morphotype	7.00	.0019	0.27	.7670
Density	10.00	.0002	3.13	.0504
Morphotype by Density	5.00	.0011	2.07	.0957

*Note*: Degrees of freedom: Morphotype (2); Density (2) Morphotype by Density (4); Residual (64).

## 4 DISCUSSION

This study provides, for the first time, information on burrowing behavior of the freshwater mussel *C. ambigua* using two distinct morphotypes, which are shaped by hydrodynamics and/or sexual dimorphism (Simeone et al., 2022). In our experiment, *C. ambigua* presented different patterns of shell exposure linked with shell shape and density. Although statistically significant differences were not found, both morphotypes tended to carry out greater horizontal movements at higher densities. Mussels may burrow to avoid predation or due to desiccation and/or flooding (Lymbery et al., 2021). Transporting mussels from their natural habitat to a manipulated one may have increased the stress of *C. ambigua* specimens. However, the experiment was carried out after a period of adaptation to reduce the effects of stress. *Castalia ambigua* has a wide distribution in South America (Pereira et al., 2014); therefore, our results may be used as a baseline to understand its burrowing behavior in other regions, taking into account potentially different shell morphologies.

**FIGURE A1:**
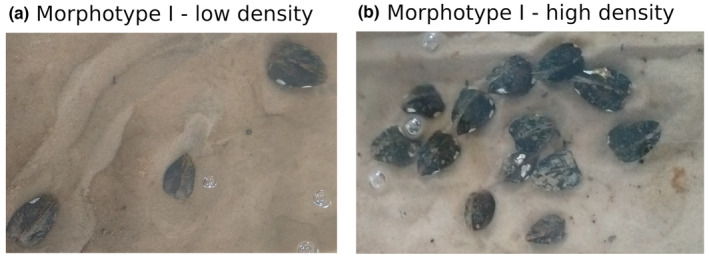
Mussel trials with 4 (a) and 16 (b) specimens of *Morphotype I*, showing the aggregation pattern observed in *C. ambigua* during the experiments.

The *C. ambigua Morphotype I* with a more elongated shell, remained less exposed in the lower density treatment (4 mussels). In this combination, all mussels remained with only the posterior margin above the sediment. Burrowing is an important behavioral response of many mussel species to persist during periods of high flow (Lymbery et al., 2021; Sansom et al., 2022). *Morphotype I* is especially associated with high hydrodynamics (Simeone et al., 2022), suggesting that their burrowing behavior may be linked with their functional morphology (Hernández et al., 2021). For example, more elongated shells may allow mussels to remain buried more deeply and yet maintain their filtration apertures extended close to the sediment surface, facilitating filtration in more hydrodynamic areas. Interestingly, *Morphotype I* presented more exposed shells in treatments with higher densities (8 and 16 mussels). In these combinations, we observed more horizontal movements and a tendency for aggregation (Figure A1). These patterns are similar to those observed in all density treatments for *C. ambigua Morphotype II* with rounded shells that are mainly found in low hydrodynamic energy habitats. Extrapolating to the natural habitat in the Caeté River, the densities of 8 and 16 mussels used in our trials are equivalent to 32 and 64 mussels per m^2^ (Simeone et al., 2021b). We suggest that patches with high mussel densities may stabilize the substrate (Christian et al., 2020; Daniel et al., 2018), reducing the effect of hydrodynamics around their shells (Randklev et al., 2019). May and Pryor (2016) found similar results, with dense mussel beds stabilizing the riverbed in high hydrodynamic energy habitats. Remaining buried for long periods may prevent feeding from the water column (Curley et al., 2021, 2022). Therefore, a stable riverbed environment, likely created by the aggregation of *C. ambigua*, may allow the species to be more exposed, facilitating the filtration of food particles. Similar results are observed for sculptured mussels in temperate rivers, which remain more exposed in habitats with lower hydrodynamic energy (Hornbach et al., 2010; Newton et al., 2015).

We suggest that *C. ambigua* aggregation may be associated with intrinsic factors of the species. However, there is a lack of information about the factors that initiate mussel aggregation (Archambault et al., 2014). Studies suggest that this pattern may be associated with reproduction (Amyot & Downing, 1998; Kemble et al., 2020; Schwalb et al., 2015). For example, mussels may move horizontally and aggregate during the spawning period (Schwalb & Pusch, 2007). This behavior facilitates the capture of sperm by females that fertilize the eggs in the suprabranchial chamber (Kemble et al., 2020; Schwalb et al., 2015). *Castalia ambigua* can remain reproductively active throughout the year (Vale et al., 2004). However, we may not directly infer that the aggregation pattern is linked to reproduction, since our experiment was carried out in short, experimental trials in the laboratory. Another hypothesis is that aggregation may be associated with mussel feeding, in order to better access food in areas with higher concentrations of seston (Schwalb & Pusch, 2007).

We also suggest that differences in burrowing behavior of *C. ambigua* may be associated with sexual dimorphism (Schwalb et al., 2015). For example, Simeone et al. (2022) demonstrated that specimens with elongate posterior margin and laterally compressed shells (*Morphotype I* in our study) were males. In contrast, specimens with rounded anterior–posterior margins and laterally inflated shells (*Morphotype II*) were females. We did not identify the sex of the mussel specimens used in this study because we returned them to their original habitat, since populations of *C. ambigua* are drastically declining in the Caeté River (Simeone et al., 2021a). However, the association of males and females with *Morphotype I* and *II*, respectively, is consistent (Simeone et al., 2022), suggesting that differences in burrowing behavior may also be linked to sex. This is very interesting, because in habitats with high hydraulic energy both morphotypes have similar shapes: elongate posterior margin with the anterior margin laterally inflated (Simeone et al., 2022). Therefore, we hypothesize that both sexes would present a similar burrowing behavior in areas of high hydrodynamics.

The biomechanics of mussel burrowing may influence ecosystem processes in rivers (Allen & Vaughn, 2009), since their burrowing may mix sediments increasing oxygen inputs (Boeker et al., 2016) and the release of nutrients to the water column (Schwalb & Pusch, 2007). In our study, *C. ambigua* carried out more horizontal movements and are more exposed at the sediment surface at high densities, which may increase bioturbation (Vaughn & Hakenkamp, 2001). This behavior may directly affect associated macroinvertebrates, such as Chironomidae that generally occupy the infaunal layer of the riverbed (Simeone et al., 2021a).

In summary, the shell exposure and horizontal behavior observed in our study may be species dependent, since burrowing patterns may differ between mussel species (Allen & Vaughn, 2009). *Castalia ambigua* may vary in shell shape due to sexual dimorphism and effects of habitat hydrological variability (Simeone et al., 2022). Thus, additional studies using other mussel species belonging to the families Hyriidae and Mycetopodidae and the inclusion of different morphotypes are important, since the behavior of these mussels in the sediment may provide useful information on their functional roles in the river (Schwalb & Pusch, 2007).

## AUTHOR CONTRIBUTIONS

**Gisele do Carmo Reis:** Conceptualization (equal); investigation (equal); methodology (equal); writing – original draft (lead); writing – review and editing (equal). **Diego Simeone:** Conceptualization (equal); investigation (equal); methodology (equal); writing – review and editing (equal). **Colin Robert Beasley:** Conceptualization (equal); formal analysis (lead); methodology (equal); writing – review and editing (equal).

## FUNDING INFORMATION

This project did not receive any funding for the research itself.

## CONFLICT OF INTEREST STATEMENT

The authors declare that they have no conflicts or financial interest.

## Data Availability

Data associated with this manuscript are available on Dryad (https://doi.org/10.5061/dryad.bzkh189ft).
